# A Hybrid Deep Learning System for Real-World Mobile User Authentication Using Motion Sensors

**DOI:** 10.3390/s20143876

**Published:** 2020-07-11

**Authors:** Tiantian Zhu, Zhengqiu Weng, Guolang Chen, Lei Fu

**Affiliations:** 1 College of Computer Science & Technology, Zhejiang University of Technology, Hangzhou 310023, China; ttzhu@zjut.edu.cn (T.Z.); derisweng@163.com (Z.W.); 2 Department of Information Technology, Wenzhou Polytechnics, Wenzhou 325035, China; 3 School of Management, Zhejiang University, Hangzhou 310023, China; 4 College of Mechanical Engineering, Zhejiang University of Technology, Hangzhou 310023, China; fulei@zjut.edu.cn

**Keywords:** mobile authentication, VMD, CNN, SVM, semi-supervised learning

## Abstract

With the popularity of smartphones and the development of hardware, mobile devices are widely used by people. To ensure availability and security, how to protect private data in mobile devices without disturbing users has become a key issue. Mobile user authentication methods based on motion sensors have been proposed by many works, but the existing methods have a series of problems such as poor de-noising ability, insufficient availability, and low coverage of feature extraction. Based on the shortcomings of existing methods, this paper proposes a hybrid deep learning system for complex real-world mobile authentication. The system includes: (1) a variational mode decomposition (VMD) based de-noising method to enhance the singular value of sensors, such as discontinuities and mutations, and increase the extraction range of the feature; (2) semi-supervised collaborative training (Tri-Training) methods to effectively deal with mislabeling problems in complex real-world situations; and (3) a combined convolutional neural network (CNN) and support vector machine (SVM) model for effective hybrid feature extraction and training. The training results under large-scale, real-world data show that the proposed system can achieve 95.01% authentication accuracy, and the effect is better than the existing frontier methods.

## 1. Introduction

With the rapid development of mobile communication technology and the Internet, mobile devices came into being and entered people’s lives. There are many types of mobile devices, including not only common smartphones, but also multifunctional smartwatches, fitness trackers, medical monitoring equipment, and augmented reality glasses. CCS Insight forecasts that 5G-enabled phones will reach sales of 210 million units in 2020, a tenfold increase compared with 2019. In 2024, sales of 5G phones are projected to hit 1.15 billion units, accounting for 58% of all mobile phones sold that year [1].

Mobile devices have various innovative functions, which can meet the requirements of personalized customization, thereby greatly improving the quality of user’s daily lives. As the diversity of mobile device applications increases, more and more users store personal private information locally. To prevent illegal access to the private information stored on the mobile device, it is urgent to design a suitable and robust authentication mode to protect the user’s privacy according to the mobile device’s own hardware, software features, and application scenario characteristics. The evolution of mobile user authentication methods can be roughly divided into three stages: The first stage is represented by knowledge-based authentication methods, which requires users to explicitly enter authentication information, such as passwords, patterns, etc. This method only verifies whether the user has correctly entered the account credentials created in advance, but cannot determine whether the user itself is trusted. In addition, since the screens of mobile devices are generally small, the experience of some user interaction interfaces for login and input is poor. For example, if the user sets a strong password containing more than 10 characters, it will take a long time for the user to enter a long password on the mobile device before unlocking or login, which leads to poor usability and unfriendly experience. In addition, previous work has shown that knowledge-based authentication methods are not only easy to be brutally cracked [2] but also vulnerable to a series of new attacks, such as stain attacks [3,4], shoulder peeping [5,6], and sensor inference [7,8,9]. The second stage takes fingerprint authentication and face recognition as the origin. Compared with the knowledge-based authentication mechanism, the static biometric authentication method based on fingerprint and face can achieve relatively high accuracy and better respond to multiple attacks. However, such biological information collection may cause users to worry about privacy and leakage of personal information. At the same time, the security of fingerprint authentication and face recognition has also been questioned by many cutting-edge works [10,11,12,13]. Although these two commonly used methods (knowledge-based authentication and static biometric-based authentication) have been in full swing for decades, there are a series of deficiencies in usability, security, and effectiveness. Therefore, in recent years, using motion sensors such as acceleration sensors, gyro sensors, and gravity sensors as data sources, a method of authentication based on the user’s dynamic behavior, has been proposed by many researchers (e.g., gait authentication [14,15,16,17,18,19,20,21,22,23,24,25,26,27,28,29,30,31], user’s usage behavior authentication [32,33,34,35,36,37,38], and daily life behavior authentication [39,40,41,42,43,44,45,46,47,48,49,50,51]). These methods have better user experience and stronger privacy protection capabilities, laying the foundation for the development of the third phase of mobile user authentication. The effective collection and analysis of motion sensor data have become a key factor that affects the authentication ability. The existing mobile user authentication methods based on motion sensor have a series of problems, such as weak feature extraction ability, poor de-noising ability, and low authentication accuracy of the owner, which need to be further improved, mainly manifested as follows:

(1) Motion sensor data collected from mobile phones in the real-world often contain noise. At present, most of the data collection of mobile authentication work is often in a relatively closed and stable experimental environment, such as a special data acquisition laboratory. There is no need to consider the impact of noise, but once deployed into the real-world, the availability is poor. Existing work based on large-scale real data [32] only filters the data lying on flat surfaces, but in the actual environment, there are many data unrelated to authentication, such as sensor signal discontinuities, mutation, etc., so the de-noising ability is limited. Previous work [52] has envisaged the use of recursive mode decomposition methods, such as empirical mode decomposition (EMD) [53] to de-noise, which adopts an adaptive method—through recursive steps the same decomposition method successively separates different modal components in the target signal. However, EMD decomposition is easily affected by singular points, such as signal discontinuities and mutations, which makes the corresponding extreme points change greatly, resulting in distortion of the calculated envelope. Instinct mode functions (IMF) components separated on this basis will be affected by singular points, causing the IMF components to contain the mode or abrupt abnormal features of adjacent components, thus introducing new noise.

(2) In a real complex environment, the sensor data collected often lacks a trusted label. This makes supervised learning [22,24,25,27,28,32,33,35,37,38,40,41,42,47,48,49,50,51] difficult to use, while unsupervised learning [31,46] has a delay in the training phase, although a semi-supervised algorithm has been proposed in frontier work [32] to improve the accuracy, the method only uses a single classifier (SVM), and its proposed initial classifier trained with some labeled samples is a weak classifier. If the previous iteration is used continuously to classify the unlabeled data, which will cause the continuous accumulation of its own errors, then it cannot guarantee that the resulting model has high accuracy.

(3) The representation ability of a feature extracted from sensor data in existing methods is weak. The data collected from the motion sensor generally appear as a time-series signal. First, in some studies, dynamic time warping (DTW) [34] and Pearson correlation coefficient (PCC) [26] are used to directly compare the time series signal, which has some defects such as unstable signal period segmentation and fewer representative features. Second, if traditional machine learning methods are used for training, feature extraction needs to be performed initially on the original signal data. Most of the existing feature extraction methods are based on statistics and empirically extract a variety of time-domain and frequency-domain features, including average, standard deviation, maximum/minimum, etc. [22,24,25,27,28,31,32,33,35,37,38,40,41,42,46,47,51], which mainly rely on manual extraction and have limited ability to represent user behavior. Third, the existing sensor data modeling methods based on deep learning [29,30,43,44,45,48,49,50] are mostly based on the original signal data, with less consideration in signal enhancement, and a lack of deeper analysis of user behavior signal data.

In order to overcome these challenges, this paper proposes a hybrid deep learning system suitable for mobile authentication in the real-world. The system can implicitly collect motion sensor data and use VMD to de-noise and enhance the signals when users are using mobile phones, and then send them to the server for label refactoring using a cooperative semi-supervised algorithm, and then use CNN and SVM for feature extraction and model training, and finally send the trained model back to the mobile terminal for real-time authentication. Experimental results on large-scale real data show that the system can achieve satisfactory accuracy, surpassing the existing frontier work. In the actual use process, users can first use our system to perform implicit real-time user authentication when the user opens the application. If the authentication fails, further authentication methods such as fingerprints, faces, and passwords can be used to greatly improve mobile devices’ availability and ease of use.

The main contributions of our works include the following aspects:

1. A heuristic signal enhancement method is proposed, which uses variational mode decomposition to decompose the signal, so as to eliminate the influence of noise and determine the optimal number of modes K by experiments.
2. The semi-supervised collaborative training method (Tri-Training) is used to de-noise, eliminating the influence of noise in the motion sensor data collected in the real-world, and providing high-quality data for the hybrid deep learning training model.
3. Using a hybrid deep learning method, the model structure includes CNN and SVM. CNN is used to extract the mixed features of each information component processed by VMD, and SVM is used for effective model training.
4. These methods are integrated to form a system. Data from training results under the large-scale real environment show that the system proposed in this paper can achieve 95.01% authentication accuracy, and the effect is better than the existing frontier methods.

The remainder of this article is organized as follows: Section 2 describes the background of our work. Section 3 covers system design in detail. Section 4 presents the overall evaluation of our system. Section 5 surveys the relevant work and Section 6 concludes our work.

## 2. Technical Background

### 2.1. Variational Mode Decomposition

VMD is not as susceptible to singular points in the signal as EMD; VMD is an adaptive and nonrecursive method that can analyze both nonstationary and nonlinear signals [54,55]. The essence of the VMD algorithm is the process of solving the variational problem. This process includes the construction and solution of the variational problem. The variational problem is expressed as follows: the original signal f can be decomposed into multiple IMF eigenmode functions, and it is assumed the bandwidth of each modal eigenfunction is limited and has different center frequencies. On this basis, the corresponding IMF eigenmode function is searched, and the cumulative sum of the bandwidth corresponding to each IMF is required to be minimized.

To obtain the bandwidth of each mode function, VMD first utilizes the Hilbert transform to convert each mode *u_k_* into an analytical expression in a single-sided spectral domain:

(1) $$u_{k}^{+}(t)=\left(\delta (t)+\frac{j}{\pi t}\right)*u_{k}(t)$$

After the Hilbert transformation, the frequency spectrum of each mode is shifted to the baseband and the corresponding estimated center frequency is adjusted by using an exponential tuned term. Then, the bandwidth is estimated according to the Gaussian smoothness of the demodulated signal by utilizing the squared L2-norm of the gradient [56]. Thus, the VMD process is realized by solving a constrained variational problem [57] and the model is expressed as follows:

(2) $$\underset{\left\{u_{k}\},\{\omega_{k}\right\}}{\min}\left\{\sum_{k=1}^{K}{\left\|\partial \left[\left(\delta (t)+\frac{j}{\pi t}\right)*u_{k}(t)\right]e^{-j\omega_{k}t}\right\|}_{2}^{2}\right\},\;\;subject\;to\;\sum_{k=1}^{K}u_{k}(t)=f(t)$$

where *f*(*t*) is the target signal, {*u_k_*} = { *u*_1_, …, *u_K_*} represents the set of the decomposed modes, and {*ω_k_*}: = {*ω*_1_, …, *ω_K_*} represents the respective center frequencies. Then, replace the solution of the constrained variational problem with the optimal solution of the unconstrained variational problem via a quadratic penalty term and Lagrangian multipliers:

(3) $$ℒ\left(\{u_{k}\},\{\omega_{k}\},\lambda \right)=\alpha \sum_{k=1}^{K}{\left\|\partial \left[\left(\delta (t)+\frac{j}{\pi t}\right)*u_{k}(t)\right]e^{-j\omega_{k}t}\right\|}_{2}^{2}+{\left\|f(t)-\sum_{k=1}^{K}u_{k}(t)\right\|}_{2}^{2}+\langle \lambda (t),f(t)-\sum_{k=1}^{K}u_{k}(t)\rangle$$

To solve the original minimization problem, the alternate direction method of multipliers (ADMM) is adopted to determine the saddle point of the augmented Lagrangian in a sequence of iterative suboptimizations. By this process, all the mode functions are obtained and updated by Wiener filtering to tune the center frequency in the spectral domain:

(4) $$\begin{array}{l} {\hat{u}}_{k}^{n+1}(\omega )=\frac{\hat{f}(\omega )-\sum_{i<k}{\hat{u}}_{i}^{n+1}(\omega )-\sum_{i>k}{\hat{u}}_{i}^{n}(\omega )+\frac{{\hat{\lambda }}^{n}(\omega )}{2}}{1+2\alpha {\left(\omega -\omega_{k}^{n}\right)}^{2}}\;\;\;\;\;(\omega >0)\\ \omega_{k}^{n+1}=\frac{\int_{0}^{\infty }\omega {\left|{\hat{u}}_{k}^{n+1}(\omega )\right|}^{2}d\omega }{\int_{0}^{\infty }{\left|{\hat{u}}_{k}^{n+1}(\omega )\right|}^{2}d\omega } \end{array}$$

The center frequency *ω_k_^n^* is calculated from the weighted center of each mode in the spectral domain; the center represents the frequency of the least squares linear regression of the instantaneous phase.

### 2.2. Semi-Supervised Training

#### 2.2.1. Co-Training

Semi-supervised learning can learn from a small amount of labeled sample data and a large number of unlabeled data, thereby improving data utilization and system performance, and reducing training costs. Collaborative Training (Co-Training) has been a popular semi-supervised learning algorithm since it was proposed by Blum et al. [58], and has become a research hotspot in the field of machine learning and pattern recognition. In the process of Co-Training, it is often necessary to establish two or more classifiers for effective cooperation. In the process of two classifiers Co-Training, the new labeled data generated by a single classifier will be used as labeled data to enter the next iteration training process of another classifier.

The basic idea of Co-Training follows. Determine two fully redundant and independent feature sets F1 and F2 on the entire data set D; these two features can be classified independently and effectively. Record the labeled parts of the data set as L and the unlabeled part as U. During training, the first step is to train two classifiers C1 and C2 on F1 and F2 of L, respectively. In the second step, u samples are randomly selected from the unlabeled data set U and put into the set U’, and all elements in U’ are labeled with C1 and C2. In the third step, m positive samples and n negative samples with the highest reliability are taken from the results of the two classifiers and put into L. In the fourth step, 2p + 2n data are selected from U and added into U’. Repeat the third and fourth steps until the number of samples reaches the required size, and the resulting L is a large number of labeled sample sets.

#### 2.2.2. Tri-Training

In general, it is difficult for data set D to meet the requirement of having two fully redundant and independent feature sets. Zhou et al. proposed the Tri-Training strategy [59], which requires neither fully redundant and independent feature sets, nor the use of different classification algorithms. Tri-Training ensures the difference between the classifiers by training on the differential data subsets extracted from the original data set.

The basic idea of Tri-Training follows. First, the labeled data set L can be repeated by sampling (bootstrap sampling) to obtain three labeled training sets L1, L2, and L3, and then generate a classifier from each training set, recorded as C1, C2, and C3. These three classifiers are used to generate pseudolabeled samples in the form of “minority obeying majority.” For example, if C1 and C2 predict a certain unlabeled sample s as a positive class and C3 predicts it as a negative class, then s is provided as a pseudolabeled positive sample to C3 for learning. Specifically, if two classifiers have the same prediction for the same unlabeled sample, the sample is considered to have higher label confidence and is added to the labeled training set of the third classifier after labeling.

However, when used in the real-world, the prediction results of the classifier may be wrong. At this time, from the perspective of “minority” classifiers, it received samples with “marked noise”. Based on the classification noise process introduced by Angluin and Laird [60], Zhou et al. [59] have derived the condition in the form of “minority obeying majority”. In each round of learning, we only need to judge whether the condition is true, and then we can decide whether to update the classifier based on the pseudolabel samples. Intuitively, this result shows that the negative impact of the introduced noise can be offset by the benefits of using a large number of unlabeled examples, and the accumulated marked noise under certain conditions can be compensated by using a large number of unlabeled data.

## 3. System Design

This section mainly introduces the design process of the system. First, we describe the overall framework of the system. Second, we introduce the data collection strategy and method. Third, we introduce how to use VMD to decompose and de-noise the signal. Fourth, we introduce the use of Tri-Training for label refactoring. Fifth, we describe the hybrid training architecture and method based on CNN and SVM, including how to conduct real-time authentication.

### 3.1. System Framework

**Figure 1:**
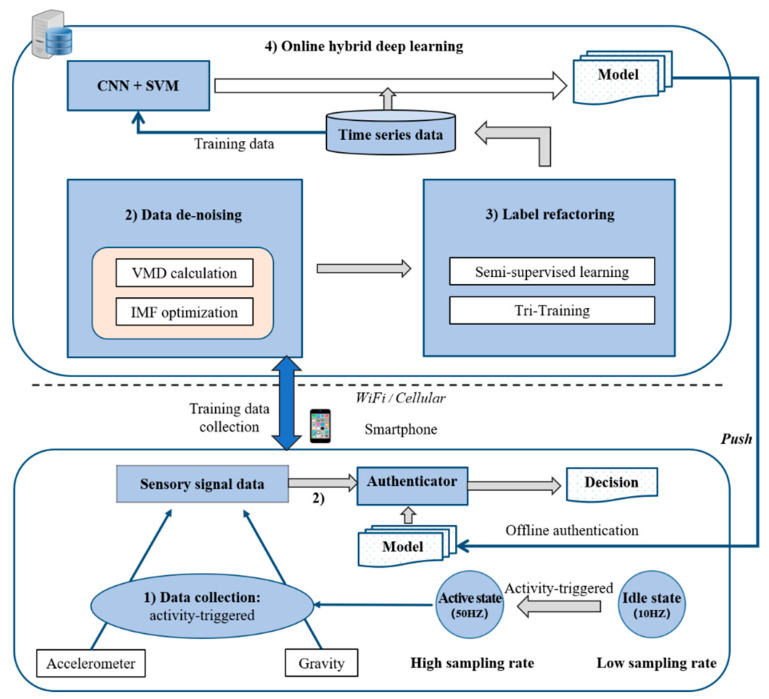
System architecture, including activity-triggered data collection in the client side, data de-noising, and label refactoring online learning with the enhanced semi-supervised model on the server side (convolutional neural network (CNN) and support vector machine (SVM)).

The overall framework of the system is shown in Figure 1, which shows that simpler calculation tasks, such as data collection and data de-noising, are performed on the mobile phone, while tasks with relatively complex calculations, such as label refactoring and online hybrid deep learning model training, are performed on the server. In addition, in order to ensure the real-time authentication, the final training model (including CNN and SVM) will be pushed to the local for offline real-time authentication, to ensure that the smartphone can be used normally when the network signal is poor or there is no network.

In the training phase, when the user first opens the mobile application (this behavior can represent a user’s behavior patterns [32]), the acceleration and gravity sensor three-axis values of the smartphone are collected as the original time series signal data. Among them, acceleration sensors are used for authentication, and gravity sensors are used for data de-noising. Then, these data will be uploaded to the server via WiFi or cellular network for de-noising and signal enhancement. Then, the data after decomposition and de-noising will be sent to the label refactoring module for labeling operation. Eventually, the labeled data will be fed into the hybrid deep learning architecture based on CNN and SVM for model training, and the optimal model suitable for real-time authentication of the host will be obtained.

In the real-time authentication phase, the motion sensor data collected will be locally de-noised and the signal enhanced, and the resulting output signal data will be sent to the fully trained CNN architecture to extract features, and the optimized SVM model will be used for real-time authentication.

### 3.2. Data Collection

Data collection is the first step in mobile authentication. What data to collect and how to collect data are the key factors that affect the final authentication accuracy. Previous research work [32] has proposed a method for real-time mobile authentication without user consciousness, which mentions how to effectively collect motion sensor data representing the user’s habits in the process of using mobile phones. Inspired by [32], this paper collects data when the user opens the mobile application. 

We develop an Android-based application and start the background service to monitor the application running in the foreground in real-time. When the following two requirements are met, data collection will be started. First, the screen of the smartphone is on. Second, the application in the foreground is switched. These two conditions indicate that the user is opening a certain application and using the mobile phone, which was proved to be a valid user mode for mobile authentication in [32]. In the actual collection process, the data collection time lasts 3 s. If the user uses the mobile phone for less than 3 s (the mobile phone screen is off during the collection process), the collection is terminated.

When judging the application of the foreground, Android provides two APIs: getRunningAppProcesses() and getRunningTasks(), to retrieve the current application running in the foreground. However, starting with Android 5.0, those APIs are deprecated. To preserve the portability of our system on the fragmented Android devices, we invoke the system command-line tool “ps” and implement a parser to map the ID of a running application (i.e., pid) to the application name on Android 5.0 and Android 6.0. Since Android 7.0, Android has locked down the permissions of/proc, and we cannot get the running process via ps. Instead, the list of running apps can be alternatively fetched by using UsageStatsManager [61] on Android 7.0+. Our implementation allows us to intercept the active applications properly on all the existing versions of Android. The summary of our implementation is shown in Table 1.

In order to save power, the services running in the Android background will collect and judge data under different frequencies, which can be divided into the idle state and the active state. Generally, the background data sampling frequency is idle, which is 10 Hz. When these two data collection conditions are met at the same time, the data collection frequency will jump from the idle state to the active state, and the sampling rate is 50 Hz.

### 3.3. Data De-Noising

Data de-noising includes two aspects. One is to remove sensor data (invalid data) that is irrelevant to the user’s mobile phone habits. The other is to eliminate the effects of singular values, such as signal discontinuities and mutations in motion sensors.

The first type is invalid data that cannot represent the users’ usage behavior, and this part of the data is not related to our authentication. When collecting data in actual scenarios, it is usually impossible to guarantee that the user always holds the mobile phone in daily use. For example, users can place the device on a desktop for interactive operation. In this case, even if the two collection requirements are met (the mobile phone screen is on and a new application is running in the foreground), the data collected still cannot effectively reflect the differences in usage patterns between different users. By asking 20 volunteers to pick up the phone and place it on a fixed surface, we get the boundaries of the gravity sensor readings on three dimensions by minimizing the errors of device placement prediction; if the readings of gravity sensor meet −1.5 < X_gr_(k) < 1.5, −1.5 < Y_gr_(k) < 1.5, and 9 < | Z_gr_(k) | < 10 simultaneously, we regard it as invalid data and remove it. Here, X_gr_(k), Y_gr_(k), and Z_gr_(k) represent values of the three axes of the gravity sensor x, y, and z at time k.

In the second type, for the recognition of different usage patterns and authentication of different mobile users, it is essential to utilize a classifier to analyze the signal obtained from the current sensors. However, it is difficult to apply the time-series signal directly for common classification methods since it may contain noise in the real-world scenario.

In order to carry out subsequent effective feature extraction (including statistical feature extraction during the label refactoring stage and CNN feature extraction during hybrid deep learning training), we need to de-noise and enhance the data first. VMD is utilized to decompose the signal obtained into several IMFs for potential feature extraction, as described in previous work [54]. The submode decomposed by VMD contains a specific spectrum, which can accurately trace the signal changes. Thus, signal decomposition can effectively eliminate the impact of noise and separate useful components in high-level modes. In VMD, the decomposition results depend on the mode number, which is defined as K. Another parameter α represents the data-fidelity constraint, which influences the tightness of the band-limits. It is also important in the decomposition. Parameter α is selected as 2500, based on the experimental trial. The value of K will be given through detailed experiments described in Section 4.

### 3.4. Label Refactoring

The motion sensor data collected from the smartphone in the real-world may not be the data used by oneself. For example: In a class, Bob may lend his mobile phone to Alice. If it happens to be in the training stage of Bob’s model, Alice’s usage habits will become a mislabeling effect and enter the classifier, and ultimately affect Bob’s model accuracy. In this regard, after obtaining de-noising and enhanced signal data, we use Tri-Training for label refactoring to eliminate the impact of other (mislabeled) data on the Bob model as much as possible.

**Figure 2:**
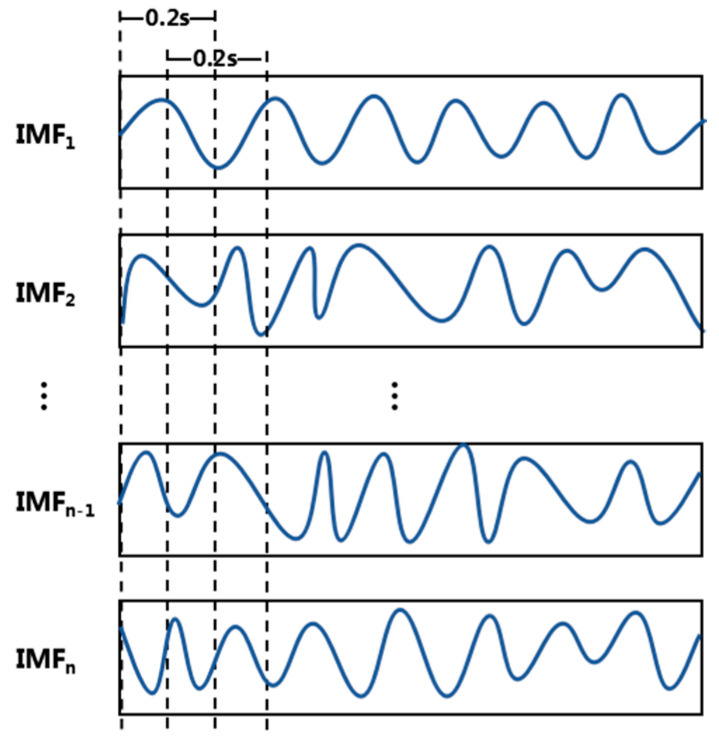
Schematic diagram of the sliding window (instinct mode functions (IMF)).

We use three different classifiers (decision tree, logistic regression, naive Bayes) to carry out label refactoring comprehensively. The specific steps follow:

(1) Feature extraction. Since the inputs of these three classifiers cannot be original time series data, it is necessary to segment the IMFs signal first and extract the features of each segment. During segmentation, we use a sliding window to slice the data into 0.2 s, and 50% overlap each slice front and back, as shown in Figure 2, (the front and back windows are shown by the dotted lines in the figure have a 0.1 s overlap).

After that, we integrated the previous work [22,24,25,27,28,31,32,33,35,40,41,42,46] and selected nine types of features, including RMS, standard deviation, variance, range, skewness, kurtosis, mean, average deviation, and permutation entropy. We denote the ith value of the feature vector as ${ℱ}_{i}=\left\{F_{1i},F_{2i},\ldots ,F_{pi}\right\}$, which includes P features, with feature extraction of 0.2 s data in each sliding window. All the features and the corresponding expressions are listed in Table 2. For each IMF, we get 29 feature vectors (3/0.1 − 1 = 29) because we utilize the sliding window design with 50% overlap.

(2) Tri-Training. In the Tri-Training phase, the original data set D (10-day data set, which will be explained in Section 4) is divided into three parts: labeled data set L, potential mislabeled data U, and verification data set V. We assume that the person who uses the mobile phone at the beginning is himself, so we put the data of the first two days into L, and the data in the middle six days may contain other people’s mislabels and put them into U. In the last two days, the data representing the user’s own test data are put into V.

We perform bootstrap sampling on the labeled data set L to obtain three labeled training sets Lc, Ll, and Ln, each set contains one-third of the total L. Then, we generate a classifier from each training set, denoted as Cd, Cl, and Cn. These three classifiers are used to generate pseudolabeled samples in the form of “minority obeying majority”. For example, if Cd and Cl predict a certain unlabeled sample s as a positive class, and Cn predicts it as a negative class, then s is provided as a pseudolabeled positive sample to Cn for learning. Specifically, if two classifiers have the same prediction for the same unlabeled sample, the sample is considered to have higher label confidence and is added to the labeled training set of the third classifier after labeling. At the same time, we use the conditions required by the “minority obeying majority” [59] to eliminate the classification error. Due to a large number of samples in this paper, the negative effects of the noise can be offset to some extent [59]. Finally, we take the intersection of Lc, Ll, and Ln as the final labeled training data set L’.

Here, we choose the binary classification, which can better represent the different mobile device usage patterns of the device owner and others. In the process of using decision tree, logistic regression, and naive Bayes classifiers, we need to obtain a certain proportion of other people’s data as a counterexample. In this paper, we use the strategy of strategic sampling in [32] to extract the most representative counterexample data samples from the massive set of others’ data.

### 3.5. Hybrid Training Based on CNN and SVM

The hybrid training based on CNN and SVM mainly includes two parts. The first part uses CNN architecture for feature extraction. The second part uses SVM to classify the extracted features.

**Figure 3:**
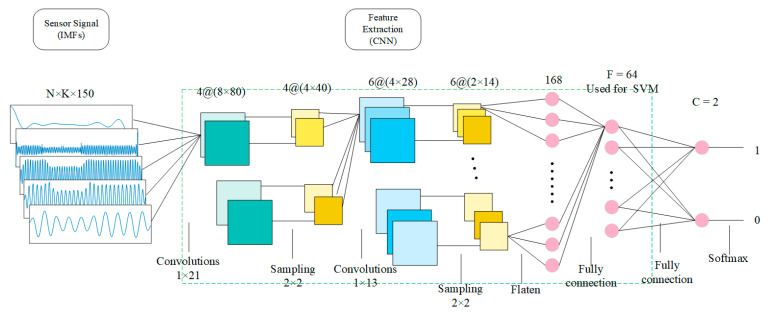
CNN extraction structure (used to extract features).

The structure of CNN is shown in Figure 3. The structure and description of each layer follow:

Input layer. The IMF signal obtained by VMD decomposition and label refactoring will be used as the input of CNN. Since the final output is label 1 (representing owner) or label 0 (representing others), CNN’s input must contain both owner’s data and others’ data, where K represents the mode number determined by VMD and N represents the IMF number (including owner and others).

Convolutional layer. The parameters of this layer are obtained by tuning. The convolutional layer implements a one-dimensional kernel (1 × 21 samples and 1 × 13), performing filtering of the input and processing each input vector separately. The activation functions are Relu.

Sampling layer (pooling layer). The parameters of this layer are obtained by tuning. Maximum pooling is applied to the output of convolutional layers to further reduce their dimensionality and increase the spatial invariance of features.

Tiled layer. The neuron is tiled to connect to the next fully connected layer.

Fully connected layer. The parameters of this layer are obtained by tuning. In the fully connected layer, each output neuron of the last layer is connected to all input neurons of this layer (weights are not shared).

Softmax layer. The classification results of the training stage were obtained.

After training the parameters of the CNN network, each time a set of data representing a user’s behavior (K × 150) is input, 64 feature values can be obtained for SVM training.

After the training, the CNN network parameters and SVM model will be pushed to the mobile phone. During the authentication phase, every three seconds of the user’s behavior patterns are collected, the invalid data will be filtered by the de-noising method, and the VMD is used to eliminate the effects of noise. Then it is fed into the CNN architecture to obtain a feature vector composed of 64 features. Finally, it is sent to the SVM model representing the owner for real-time authentication. By setting the classification threshold θ, if the output is greater than θ, it is the owner; otherwise, it is others.

## 4. Experimental Evaluations

### 4.1. Data Set

In this paper, we have two data sets. The first data set is used to evaluate the overall performance of our system, and the second data set is used for verifying how many training samples are enough for model training. For the first data set, we collected large-scale unlabeled data in a real unsupervised environment, which comes directly from an Internet company with millions of users in China. For ethical considerations, all participants were actively informed of the purpose of data collection, and given the option to opt-in or opt-out. In the end, we collected data from 1513 users using 10 days of raw data. In this paper, a total of 283,133,354 pieces of raw sensor data were collected. After data preprocessing, 283,006,659 pieces were found to be valid, and the average number of effective records per user was 187,050 (about 37,409 samples, 187,050/50/0.1 − 1 = 37,409).

In the label refactoring phase, decision tree, logistic regression, and naive Bayes classifiers are used to divide the labeled data set into a training set and a test set during training, and the ratio is 4:1. The ratio of positive samples to negative samples is 1:5, and the data volume of positive samples and negative samples in the test stage is also extracted according to the same ratio distribution.

In the hybrid training phase based on CNN and SVM, we divide the labeled data set L’ into a training set and a test set after Tri-Training, with a ratio of 4:1. The ratio of positive samples to negative samples is 1:5, and the data volume of positive samples and negative samples in the test stage is also extracted according to the same ratio distribution.

For the second data set, we experimented with 106 people based on the data collected within one week from another large Internet company with labels, and the average number of effective samples per user ranges from 1024 to 20,132, depending on the frequency of the usage.

For all the data sets, the data collection frequency is 50 Hz, and each data collection lasts 3 s. In order to uniquely mark each user, we will acquire the international mobile equipment identity (IMEI) while collecting data and use IMEI to distinguish different users on the server side.

### 4.2. Evaluation Index

In terms of accuracy, we define the following evaluation matrix. True positive (TP), the owner is accurately marked. False positive (FP), others are marked as owner. True negative (TN), others are accurately marked. False negative (FN), the owner is marked as others. In terms of classification accuracy, the following indicators are used in this chapter:

Powner=TP/(TP+FP)

Rowner=TPR=TP/(TP+FP)

Fowner=2∗Powner∗Rowner/(Powner+Rowner)

Pother=TN/(TN+FN)

Rother=TN/(TN+FP)

Fother=2∗Pother∗Rother/(Pother+Rother)

FPR=FP/(TN+FP)

Accuracy=(TP+TN)/(TP+FP+FN+TN)

Powner, Rowner, and Fowner represent the precision, recall, and F1-score of user owners, respectively. Pother, Rother, and Fother represent the precision, recall, and F1-score of other users, respectively. In addition, we use the receiver operating characteristic (ROC) curve to represent the overall authentication performance of the system. The ROC curve shows the relationship between the true positive rate (TPR) and false positive rate (FPR) at different classification thresholds θ. In particular, the area under curve (AUC) is used to represent the area enclosed by the ROC curve and the coordinate axis.

### 4.3. VMD Effect Analysis

**Figure 4:**
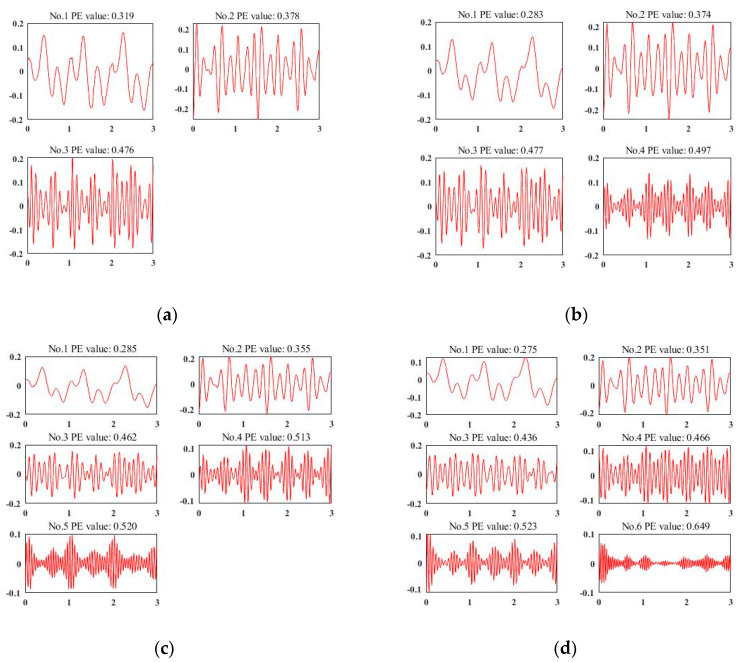
Sensor signal decomposition results by variational mode decomposition (VMD). (**a**) K = 3 by VMD; (**b**) K = 4 by VMD; (**c**) K = 5 by VMD; (**d**) K = 6 by VMD.

To deploy VMD for data de-noising and signal enhancement, the most important thing is to calculate the decomposed number K via experiment. Figure 4a–d represent a representative acceleration sample of different decomposition results of VMD when K = 3, 4, 5, and 6, respectively. According to the experience of the existing work [54], we use the permutation entropy of each IMF to judge the quality of the decomposition results. We set the threshold as 0.6 as [54] did. It can be observed that the sensor signals in Figure 4a–c are under-decomposed. Although the decomposition results are useful, they are still far from the target threshold. When K = 6, the first five IMFs are valid for our classification, while the last one can be considered as noise, which totally meets the requirements for data de-noising and signal enhancement. Finally, for each sensor signal, we choose K = 6 and select the first five IMFs as valid ones.

**Figure 5:**
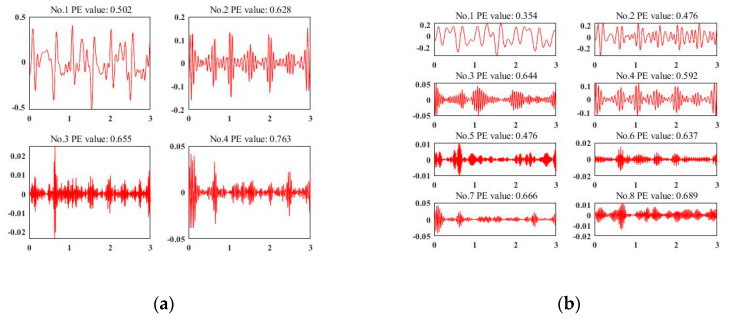
Sensor signal decomposition results by wavelet packet decomposition (WPD). (**a**) The decomposition level is 2 by WPD; (**b**) the decomposition level is 3 by WPD.

We also compare our VMD method to another famous signal decomposition method [62], called wavelet packet decomposition (WPD). Figure 5a,b presents the decomposition results by WPD when the decomposition level is 2 and 3, respectively. When K = 2, only the first decomposition result is valid (the value of permutation entropy is less than the threshold 0.6). Also, when K = 3, the valid decomposition result is less than that of VMD. VMD can perform better even if the decomposed number K is not suitable for decomposition.

### 4.4. Model Tuning

**Figure 6:**
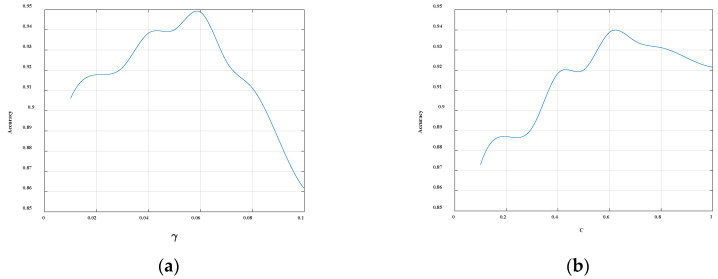
(**a**) The relationship between the parameter γ and the final classification accuracy of the model. (**b**) The relationship between the parameter C and the final classification accuracy of the model.

In order to optimize the final SVM model, we conduct the grid search to find the optimal SVM training parameters. In SVM, the two main parameters that need to be determined are C and γ, where the C is the penalty coefficient. If C becomes larger, it means that the punishment becomes larger, resulting in a situation where the model is not flexible enough and the generalization ability becomes weak. Conversely, if it becomes smaller, the punishment strength will become smaller, and the model is prone to underfitting. Another parameter γ determines the distribution of the data mapped to the new feature space. Similar to parameter C, γ that is too large will cause the model not to learn the content of the vector, but only remember the support vector itself, which leads to the reduction of generalization ability and prone to overfitting. If γ is too small, the data distribution in the new feature space will be too smooth, resulting in underfitting. In this paper, the classification threshold θ is set to 0.5, the SVM convergence coefficient is fixed at 0.01, and the parameter γ is fixed at 0.01. The changes in the parameters and the final classification accuracy of the model are shown in Figure 6. The classification effect is the best when the value of the parameter C is 0.6. Similarly, when the SVM convergence coefficient is fixed at 0.01 and the parameter C is fixed at 0.6, the changes in the parameters and the final classification accuracy of the model are shown in Figure 6. It can be seen from the figure that when the value of the parameter γ is 0.06, the classification effect is the best. In summary, C = 0.6 and γ = 0.06 are used in this paper.

### 4.5. Overall Accuracy

Under the optimal parameters, we did 10 independent repeated experiments, and the final average values of TP, FP, TN, and FN are shown in Table 3.

As can be seen from Table 3, after the label refactoring, most of the mislabeling were corrected, and the final authentication results were significantly improved. By deploying VDM, the average Powner, Rowner, Fowner, Pother, Rother, and Fother increased from 91.04%, 72.10%, 80.47%, 70.15%, 92.33%, and 79.73%, to 95.02%, 74.32%, 83.40%, 76.28%, 97.01%, and 85.41%, respectively. We also evaluate the system accuracy comparison with and without using VMD de-noising to justify the effectiveness of VMD. The results show that the IMFs decomposed by VMD can accurately trace the signal changes. Our system (labeled with VMD) reaches a final classification accuracy value of 95.01%.

As mentioned above, the ratio of positive samples to negative samples in the training set is 1:5, which shows that the trained classifier can accurately represent the mobile phone usage patterns of nonowner users, but may lose some patterns of the owner user. Considering that FP (that is, a malicious user can normally use the mobile phone of the owner user) in the authentication system is often more critical than FN (the owner user is mistaken for a malicious user), this paper tends to reduce the FP when configuring the authentication parameters. In practice, the classification threshold can be adjusted. For example, when the user operates an application with higher security, the threshold can be increased, which can improve the detection rate of malicious use of the owner’s mobile phone against others.

**Figure 7:**
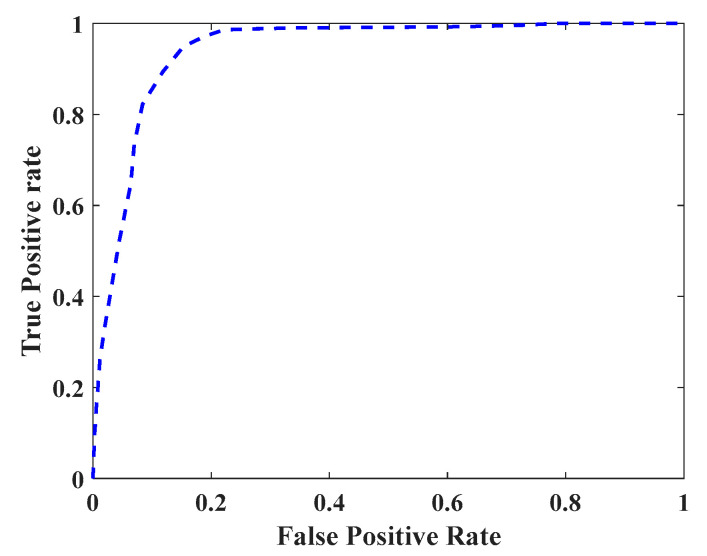
Receiver operating characteristic (ROC) curve of the system.

In order to obtain the ROC curve, we set the threshold value θ from 0 to 1, using a step size of 0.01. Under different classification thresholds θ, the average values of TPR and FPR of all users are calculated, and the resulting ROC curve is shown in Figure 7. The resulting AUC value is 0.9340. From a security point of view, an increase in FPR will cause a greater potential threat to the owner user. From the usability point of view, a reduction in TPR will lead to a reduction in user experience. Due to the high AUC, the system has sufficient optimization space to balance the security and availability in actual use.

This paper investigates the most advanced research on motion sensor-based mobile authentication in the past five years and uses large-scale data sets to test its proposed classification methods. Table 4 shows a comparison of the accuracy for the five different methods in the existing work and the methods employed in this paper.

As can be seen from the table above, the accuracy of the authentication system in this paper is higher than other methods [32,34,35,41,42]. Among them, we also use the binary-class SVM for training, and the reason why the method proposed in this paper is better than [32] is that CNN is used for feature extraction, which has a wider coverage and more representative of the user’s patterns than that of the manually extracted signs in [32]. Interestingly, the one-class SVM performs worse than the binary-class SVM used in this article. The main reason is that the characteristics of different users’ mobile phone behaviors may be similar in multidimensional space, and the boundary of this approximate behavior cannot be well represented by one-class SVM.

**Figure 8:**
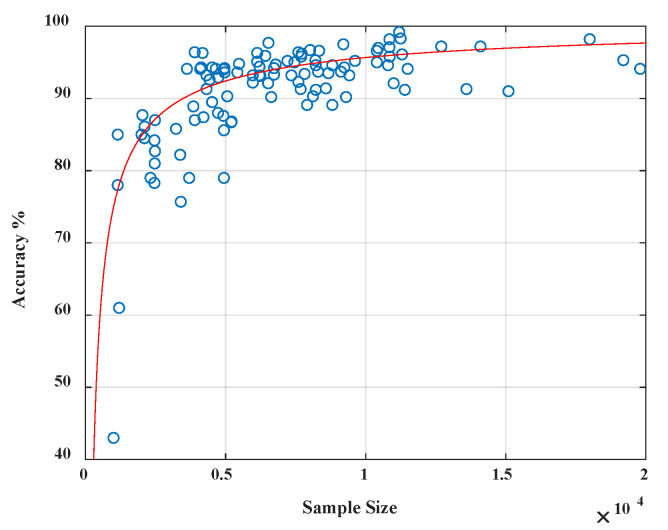
Sample size vs. accuracy showing that classification accuracy improves drastically when the sample size (0–4000) increases.

Also, it is necessary to evaluate the training sample size’s impact on system accuracy since it is possible that not every user would have enough training sample to begin with. We experimented with 106 people based on the data collected within seven days. The classification model is constructed for each individual in the first five days, and the average number of effective samples per user ranges from 1024 to 20,132, depending on the frequency of the usage. The data from the last two days are used for testing. The accuracy for each model is evaluated in Figure 8, and it shows that the accuracy has a strong relation to the sample size of positive instances. When the sample from the authorized users is insufficient, less than 4000, the performance of our classification is less satisfied with low accuracy. However, it improves drastically when the sample size increases. We also notice that once the sample size exceeds a threshold of 4000, accuracy will not improve by simply adding more samples. Since we have 37,409 data samples per user in the 1513 data set, the sample size (37409 × 0.2 = 7482 > 4000) is quite sufficient in our experiment.

### 4.6. Anti-Attack Ability

In this section, we mainly discuss the impact of imitation attacks on our system in order to verify the robustness of our system in real-time mobile user authentication. Imitation attack refers to an attacker bypassing the authentication mechanism by observing the equipment usage pattern of the owner user and imitating the behavior of the owner user.

We invited 20 participants to conduct related experiments. First, select a model that one participant trains to represent mobile owner behavior patterns. The remaining 19 participants call the authentication module 100 times by observing the way the first participant uses the mobile phone and imitating his holding gesture. Among them, 29 data samples are generated for each imitation, and these samples are sent to the model for verification. After calculation and averaging, the probability that the mobile authentication system can successfully resist imitation attacks is 99.90%.

### 4.7. Overhead

In order to meet the real-time authentication, we conducted real-time detection of energy consumption tests, including mobile phone battery consumption, CPU usage, memory usage, and real-time authentication delay.

On the client side, we use the open source tool Emmage [63] to test the battery consumption, CPU usage, and memory usage of the mobile phone while the application is running. Table 5 shows the test results. In terms of battery consumption, this paper allows a participant to continue using the authentication service on a smartphone for three hours, including data collection and offline real-time authentication. On three different Xiaomi phones (MI 8), the battery consumption per hour is about 1% of the total power of the mobile phone, because users do not frequently open or switch applications under normal circumstances.

In addition, we also test the time-consuming authentication of the client. The following tasks are carried out on the above three smartphones (MI 8): data collection, data de-noising, feature extraction, and authentication, repeated 1000 times. The average time consumption of each stage is listed in Table 6. It can be seen that the entire process is completed in 3201.08 ms. Data collection takes up most of the time, and the time for data preprocessing, feature extraction, and authentication can be completed in about 20 ms, which meets the real-time authentication needs in real scenarios.

## 5. Related Work

In recent years, there has been much work [14,15,16,17,18,19,20,21,22,23,24,25,26,27,28,29,30,31,32,33,34,35,36,37,38,39,40,41,42,43,44,45,46,47,48,49,50,51] using the motion sensor for mobile authentication. However, the existing methods are more or less flawed. In this section, we will explain the existing methods and highlight the innovation of our work from the aspects of de-noising ability, availability, and feature extraction coverage.

De-noising ability. Most of the previous user authentication studies [22,23,24,25,26,33,34,35,36,37,38,39,40,41,42,47,48,49,50,51] considered motion sensors ideally error-free during data collection and they had never taken the noise impact of the hardware into account, which would lead to the difficulty in fitting the model and thus affecting prediction accuracy. Existing work based on large-scale real data [32] only filters the data lying on a flat surface, but in the actual environment, there are many data that are not related to authentication, such as sensor signal discontinuities and mutations, so the de-noising ability is limited. [52] envisaged the use of recursive modal decomposition methods such as EMD [53] de-noising, which adopts the adaptive method by recursion of the same decomposition method, successively separating different modal components in the target signal. However, EMD is easily affected by singular points such as signal discontinuities and mutations, which makes the corresponding extreme points change greatly, resulting in distortion of the calculated envelope. The IMF components separated on this basis will be affected by singular points, causing the IMF components to contain the modal or abrupt abnormal features of adjacent components, thus introducing new noise. Different from the above work, this paper uses the VMD algorithm for effectively de-noising, and through experimental analysis determines the optimal number of modes.

Usability. In a complex real environment, the sensor data collected often lacks a trusted label, which makes the supervised learning algorithm [22,24,25,27,28,32,33,35,37,38,40,41,42,47,48,49,50,51] difficult to use, while the unsupervised method [31,46] has a delay in the training phase. For example, Zhu et al. [33] simultaneously used continuous time-domain data collected by mobile device acceleration sensors, gyroscope sensors, and magnetic sensors to model owner features, and the recognition rate of the owner users reached 75%. However, the test cycle of this method needs to last for 24 h, which makes real-time detection impossible in the real-world. At the same time, this method cannot handle unlabeled data in complex environments. Although [11] proposed an unsupervised learning algorithm to correspond to unlabeled data, the parameter adjustment of the unsupervised clustering algorithm needs to pay a great price, and the generalization ability of the parameter needs to be verified. Although frontier work by [32] proposed a semi-supervised algorithm to improve accuracy, the method used only uses a single classifier (SVM), and its proposed initial classifier trained with some labeled samples is a weak classifier. If the previous iteration is used continuously to classify the unlabeled data, which will cause the continuous accumulation of its own errors, then it cannot guarantee that the resulting model has high accuracy. Different from the above method, this paper adopts a cooperative semi-supervised algorithm (Tri-Training) for label refactoring, so that a large number of false labels are corrected and the final classification accuracy is greatly improved.

Feature extraction coverage. The existing methods have a weak ability to represent features extracted from sensor data. The data collected from the motion sensor generally appears as a time-series signal. First, there are some studies that use dynamic time warping, Pearson coefficients, and other methods to compare time-series signals directly, which have some defects, such as unstable signal period segmentation and fewer representative features. Second, if traditional machine learning is used for training, feature extraction needs to be first performed on the original signal data. Most of the existing feature extraction methods are based on statistics, and empirically extract a variety of time-domain and frequency-domain features, including average, standard deviation, maximum/minimum, etc. [22,24,25,27,28,31,32,33,35,37,38,40,41,42,46,47,51]; these type of features mainly rely on manual extraction and have limited ability to represent user behavior. Third, the existing sensor data modeling methods based on deep learning [29,30,43,44,45,48,49,50] are mostly based on the original signal data, with less consideration for signal enhancement, and lack of deeper analysis of user behavior signal data. In this paper, VMD is used for signal decomposition, which not only eliminates the influence of noise but also enhances the signal effectively, so that the input features of the model have a wider coverage.

## 6. Conclusions

Mobile authentication is a hot topic currently, and implicit real-time authentication based on motion sensors has developed rapidly in recent years. Existing motion sensor-based authentication methods have certain deficiencies in de-noising ability, usability, and feature extraction coverage. This paper proposes a heuristic signal enhancement method that uses variational mode decomposition (VMD) to decompose the signal to eliminate the effect of noise; The semi-supervised collaborative training method (Tri-Training) is used to de-noise, so as to eliminate the influence of noise in the motion sensor data collected in the real-world. At the same time, a hybrid depth learning method is used, which uses CNN for high coverage feature extraction, and SVM for effective model training. The training results under large-scale, real-world data show that the system proposed in this paper can achieve an accuracy of 95.01%, and the effect is better than the existing frontier methods.

## Figures and Tables

**Table 1:** Android version and corresponding method for obtaining foreground applications.

SDK Version (V)	Our Methods
V < 21 (Android 5.0-)	GetRunningTasks (permission.GET_TASKS)
21 ≤ V ≤ 23 (Android 5.0 and Android 6.0)	Ps or UsageStatsManager
V ≥ 24 (Android 7.0 +)	UsageStatsManager (android.permission.PACKAGE_USAGE_STATS)

**Table 2:** Statistical parameters for feature extraction.

No.	Statistical Feature	Expression
1	RMS	$\sqrt{\frac{1}{K}\sum_{k=1}^{K}{\left[x(k)\right]}^{2}}$
2	Mean	$\sum_{k=1}^{K}\left[x(k)\right]/K$
3	Standard Deviation	$\sqrt{\sum_{k=1}^{K}{\left[x(k)-\bar{x}\right]}^{2}/\left(K-1\right)}$
4	Variance	$\sum_{k=1}^{K}{\left[x(k)-\bar{x}\right]}^{2}/K$
5	Range	max(x(k))−min(x(k))
6	Kurtosis	$\sum_{k=1}^{K}{\left[\left(x(k)-\bar{x}\right)/\sigma \right]}^{4}/K$
7	Skewness	$\sum_{k=1}^{K}{\left[\left(x(k)-\bar{x}\right)/\sigma \right]}^{3}/K$
8	Average Deviation	$\sum_{k=1}^{K}\left|x(k)-\bar{x}\right|/K$
9	Permutation Entropy	$-\sum_{i=1}^{m!}p\left(\pi_{i}\right)\log\left(p\left(\pi_{i}\right)\right)$

**Table 3:** The average classification results of 10 independent repeated experiments on 1513 users, including a comparison of accuracy with Tri-Training (labeled) and without Tri-Training (unlabeled).

Categories	Powner	Rowner	Fowner	Pother	Rother	Fother
Unlabeled without VMD	89.08%	70.35%	78.61%	68.42%	90.57%	77.95%
Labeled without VMD	93.35%	72.50%	81.61%	72.91%	94.89%	82.46%
Unlabeled with VMD	91.04%	72.10%	80.47%	70.15%	92.33%	79.73%
Labeled with VMD	95.02%	74.32%	83.40%	76.28%	97.01%	85.41%

**Table 4:** Comparison of our work with other related work.

Study	Classifier	Accuracy
Our work	CNN + SVM (binary-class)	95.01%
Zhu et al. (2019) [32]	SVM (binary-class)	94.67%
Shen et al. (2018) [42]	HMM	90.54%
Buriro et al. (2017) [35]	Random forest	92.36%
Lee et al. (2017) [34]	DTW	86.49%
Zdeňka (2016) [41]	SVM (one-class)	86.51%

Hidden Markov Model (HMM), Dynamic Time Warping (DTW).

**Table 5:** Test results of client battery consumption.

Phone No.	Battery Consumption (mAh)	Data Collection		Authentication	
		CPU (%)	Memory (MB)	CPU (%)	Memory (MB)
Xiaomi No.1	146.58/3000	1.28	14.02	9.34	22.52
Xiaomi No.2	151.01/3000	1.31	14.02	9.58	22.68
Xiaomi No.3	149.31/3000	1.29	14.03	9.40	23.08

**Table 6:** Time spent on client authentication.

Procedure	Times (ms)
Data collection	3037.20
Data de-noising	17.31
Feature extraction	135.05
Authentication	11.52
Overall	3201.08
